# Nationwide study of factors associated with public’s willingness to use home self-test kit for dengue fever in Malaysia

**DOI:** 10.1186/s12889-016-3409-y

**Published:** 2016-08-12

**Authors:** Li Ping Wong, Narges Atefi, Sazaly AbuBakar

**Affiliations:** 1 Department of Social and Preventive Medicine, Faculty of Medicine, University of Malaya, 50603 Kuala Lumpur, Malaysia; 2 Julius Centre University of Malaya (JCUM), University of Malaya, Kuala Lumpur, Malaysia; 3 Department of Medical Microbiology, Faculty of Medicine, University of Malaya, Kuala Lumpur, Malaysia; 4 Tropical Infectious Diseases Research and Educational Centre (TIDREC), University of Malaya, Kuala Lumpur, Malaysia

**Keywords:** Dengue, Home self-test kit, Willingness

## Abstract

**Background:**

As there is no specific treatment for dengue, early detection and access to proper treatment may lower dengue fatality. Therefore, having new techniques for the early detection of dengue fever, such as the use of dengue test kit, is vitally important. The aims of the study were: 1) identify factors associated with acceptance of a home self-test kit for dengue fever if the dengue test is available to the public and 2) find out the characteristics of the test kits that influence the use of the dengue test kit.

**Methods:**

A national telephone survey was carried out with 2,512 individuals of the Malaysian public aged 18–60 years old. Individuals were contacted by random digit dialling covering the whole of Malaysia from February 2012 to June 2013.

**Results:**

From 2,512 participants, 6.1 % reported to have heard of the availability of the dengue home test kit and of these, 44.8 % expressed their intention to use the test kit if it was available. Multivariate logistic regressions indicated that participants with primary (OR: 0.65; 95 % CI: 0.43–0.89; *p* = 0.02, vs. tertiary educational level) and secondary educational levels (OR: 0.73; 95 % CI: 0.57–0.90; *p* = 0.01, vs. tertiary educational level) were less likely than participants with a tertiary educational level to use a home self-testing dengue kit for dengue if the kit was available. Participants with lower perceived barriers to dengue prevention (level of barriers 0–5) were less likely (OR: 0.67, 95 % CI: 0.53–0.85, *p* < 0.001, vs. higher perceived barriers) to use a home self-testing dengue kit for dengue if the kit was available compared to those with higher perceived barriers to dengue prevention (level of barriers 6-10). Participants with a lower total dengue fever knowledge score (range 0–22) were also less likely to use a home self-testing dengue kit for dengue if the kit was available (OR: 0.75; 95 % CI: 0.61–0.91, *p* = 0.001, vs. higher total dengue fever knowledge score) compared to those with a higher total dengue fever knowledge score (range 23–44). With response to characteristics of the test kit, participants indicated that ease of usability and easy to understand instructions were the most important factors influencing the decision to use the dengue home test kit; this was followed by the price of the test kit.

**Conclusions:**

The study highlights the need for provision of information to increase knowledge about the home self-testing dengue kit. Educational interventions should target people with low educational levels, those with lower dengue fever knowledge and those with lower perceived barriers to dengue prevention.

**Electronic supplementary material:**

The online version of this article (doi:10.1186/s12889-016-3409-y) contains supplementary material, which is available to authorized users.

## Background

Dengue is of particular importance in South East Asia, the Americas and the Western Pacific where it has become increasingly endemic with epidemic outbreaks [1]. The incidence of dengue has grown dramatically around the world in recent decades. More than 2.5 billion people are now at risk of contracting dengue and the World Health Organization (WHO) currently estimates there may be 50–100 million dengue infections worldwide every year including 500, 000 dengue haemorrhagic fever cases and 22,000 deaths, mostly among children [2]. In Malaysia, dengue fever is endemic with frequent major outbreaks in urban areas [3]. The largest outbreak was seen in 1996 with 14,255 dengue cases reported and 32 deaths. The fever is the number one disease among communicable diseases in Malaysia as compared to other diseases like Tuberculosis, Malaria and HIV/AIDS in 2010 and 2011 [4]. The number of dengue cases doubled from 21,900 cases in 2012 to 49,346 cases in 2013 [3].

There is no specific antiviral treatment currently available for dengue fever and to date there is no approved vaccine for the prevention of dengue, thus treatment involves trying to relieve symptoms and treatment is mostly administered to limit multi-organ complications subsequent to severe intravascular leakages [5, 6]. An early diagnosis is nevertheless very important for efficient clinical management in order to cure or prevent life-threatening complications. In addition, accurate and early diagnosis directs clinical attention to warning signs of an evolution to severe forms and avoids unnecessary use of antibiotics [7, 8].

A range of serological and virological diagnostic methods are available for dengue fever but most of these methods require specialised laboratory equipment. Serological tests are more commonly used to diagnose dengue infections because of their ease of use compared to other techniques [9]. It is based on detecting the presence of dengue specific IgM and IgG. The major disadvantage of this method is that there must be a significant rise in the dengue specific antibody titre before it becomes detectable; this can be as early as on day 3 after the onset of the fever for secondary dengue or by day 5 for primary dengue. Suspected dengue patients who come to the hospital early, within the first 72 h after onset of fever, would not be positive for dengue [9]. Since the simple serological tests may not enable early diagnosis, other alternatives tests might be selected. Early diagnostic approaches, including virus isolation and molecular tests are expensive and require expertise to perform. Recently, an up-to-date test for early diagnosis of dengue infection, the dengue NS1 antigen detection has been proposed [10]. In most cases, the dengue NS1 protein can be detected very early during the first three days after onset of fever, in patients with primary infection. There are plans to make the test available as a home self-test kit for dengue [8] and it is hoped that self-test kit for dengue fever is soon be available for public.

Studies that look at factors associated to acceptance of using self-test kit for dengue is scarce. Various factors may lead to use of the available of the home self-test kit. For instance, enough knowledge about dengue fever may be applied in acceptance of the home self-test kit, which could decrease morbidity and mortality of dengue. It has been noted that other factors such as specificity, reliability, cost, convenience and easy to understand instructions are also important [11]. In addition, health beliefs may influence medical decision-making. As such, health beliefs related to dengue fever may likely influence uptake of a dengue self-test kit. The basic concepts of the Health Belief Model (HBM) are perceived susceptibility, perceived severity, perceived benefits, perceived barriers to taking health action, cues to action and self-efficacy [12]. A number of studies have used HBM in an attempt to understand health services’ utilisation, including screenings [13–15]. Nevertheless, HBM only has been applied in a few studies in the context of the acceptance of a home self-test kit for dengue [14].

The purpose of this study was to: 1) identify factors (demographic factors, health beliefs about dengue fever, dengue fever knowledge) associated with the perceived acceptance of a home self-test kit for dengue if the test was available and 2) to find out the characteristics of the test kits that are likely to influence the use of dengue home test kit if it becomes available. It is hoped that the findings provide insights into the potential of dengue self-test in an effort to contain and prevent dengue in dengue endemic regions.

## Methods

### Sampling frame

Interviews were conducted between February 2012 and June 2013 using a computer-assisted telephone interview (CATI) system. The telephone numbers were randomly generated by the computer from the latest electronic residential telephone directory (2012/2013) of all 13 states in Malaysia. To qualify for the telephone interview, participants had to be Malaysians, between 18 and 70 years old, have heard of dengue fever and residing in the contacted household. The computer randomly generated telephone numbers from the electronic residential telephone directory. Only one person per household was surveyed. If more than one qualified person was found in a household, one person was randomly selected using a random number table. Interviews were conducted between 5:30 pm and 10:00 pm on weekdays and from 12:00 p.m. to 7:00 p.m. on weekends or public holidays to avoid over-representation of unemployed participants. Unanswered calls were attempted at least two more times on separate days before being regarded as non-responses.

### Instrument

The questionnaire (Additional file 1) about dengue fever consisted of: socio-demographic characteristics, dengue fever knowledge, a health belief questionnaire from the HBM, perceived uptake of a self-test kit and characteristics of the test kits if one should become available.

Dengue fever knowledge consists of 44 items. For each statement, participants could choose between three response categories: 'yes', 'no' and 'don't know'. For analyses, participants were scored as 1 for a correct response and 0 for an incorrect or 'don't know' response. Several negatively worded items were reversed and re-coded during the data analysis process. Possible scores ranged from 0 to 44. Higher scores indicate greater dengue knowledge.

Belief questions were based on several HBM constructs. Severity perception regarding dengue fever consisted of two parts: 1) perceived severity and perceived susceptibility of dengue fever, where the perceived severity assesses feelings concerning the seriousness of dengue fever and 2) perceived susceptibility assesses one's subjective perception of the risk of contracting dengue fever. Perceived severity of dengue fever was measured on a scale of 0–10 with higher score indicating higher severity. Likewise, perceived susceptibility of dengue fever was measured on a scale of 0–10 with a higher score indicating higher susceptibility. Perceived barriers to dengue prevention examine perceptions of barriers to prevent dengue fever among participants. This was also measured on a scale of 0–10 with a higher score indicating greater barriers. Self-efficacy was the opinion of the respondent regarding their ability to successfully manage the dengue prevention behaviour; self-efficacy was measured by a 4-point Likert scale that ranged from 1 (strongly agree) to 4 (strongly disagree).

Perceived uptake of the dengue self-test kit consisted of three questions. First, “would you consider using a home self-test kit for dengue if the kit is available?” Second, participants were asked, “Would you be able to prick your finger to do self-testing for dengue?” Thirdly, characteristics of the test kits which influence the use of dengue test kit if available, consist of five questions; easy to obtain, easy to use and instructions easy to understand, accuracy of test kit, price and recommendation by health care providers.

Demographic questions (10-item) were asked after completion of the survey questions. To avoid the problems inherent in translation, two bilingual experts translated the instruments from English to Bahasa Malaysia, after which they were again blindly back-translated by two other bilingual experts. Content validity of the questionnaire was assessed by groups of experts to ensure that the items have acceptability content validity. The final draft version of the questionnaire was pilot tested.

### Statistical analyses

All statistical analyses were performed with the Statistical Package for the Social Sciences Version 20.0 (SPSS; Chicago, IL, USA). Non-responses and irrelevant answers were treated as missing values and therefore excluded from the analyses. Values of *p* < 0.05 were considered significant. In addition to descriptive analyses, the chi-square test was used to test the significance of differences in percentages. Multivariable logistic regression was used to identify factors associated with the intent to use the dengue home test kit if it becomes available. In the modeling strategy, the independent variables were included in the model if they had a *p* of < 0.05 on univariate analysis using the 'Enter' method.

## Result

### Participants’ characteristics

**Fig. 1:**
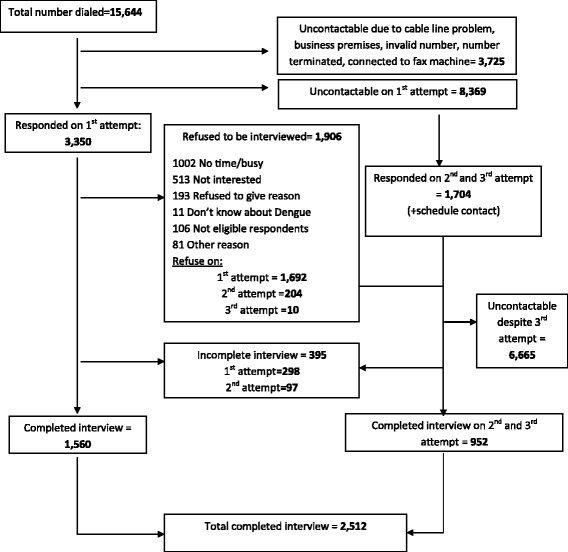
Illustration of the CATI process of the survey

Figure 1 shows the flowchart of the CATI process. A total of 15,644 call attempts were made, resulting in 2,512 responding households. The response rate computed as the number of completed interviews (2,512) divided by the number of eligible and contactable households (5,354) was 46.0 %. Participants who declined to participate in the survey were asked for their reasons for not wanting to participate. The most common reasons for not wanting to participate were “busy” (35.2 %) and “not interested” (18.0 %). A total of 11 (0.3 %) participants said that they did not know about dengue, and declined to be interviewed.

As Table 1 shows, the mean age of the sample was 40.18 (16.08) years, age range 18 to 70 years old. There were more females than males in this study. Most of the study participants had secondary school education. The majority of participants were Malay (55.5 %). Less than half of participants 31.5 % (*n* = 790) had a monthly average income below RM 2,000.

**Table 1:** Socio-demographic characteristics of participants (*N* = 2512)

Socio-demographic variables	N	%
Age (years)		
18–40	1272	50.6
>41 years	1240	49.4
Gender		
Male	717	28.5
Female	1795	64.3
Marital status		
Single	766	35.7
Ever married	1381	64.3
Ethnicity		
Malay	1394	55.5
Chinese	771	30.7
Indian	249	9.9
Others	98	3.9
Religion		
Muslim	1477	58.8
Christian	152	6.1
Buddhist	515	20.5
Others	268	14.7
Highest educational level		
No formal education	43	1.7
Primary school	353	14.1
Secondary school	1292	51.4
Tertiary education	824	32.8
Occupation		
Professional and managerial	333	13.3
Skilled worker	334	13.3
Non-skilled worker	396	15.8
Student	485	19.3
Housewife	702	27.9
Retired	205	8.2
Others	57	2.3
Monthly average household income		
Below RM1000	303	12.1
RM1001–RM2000	487	19.4
RM2001–RM3000	411	16.4
RM3001–RM4000	233	9.3
RM4001–RM 5000	109	4.3
More than RM5001	225	9
Type of house		
Flat/Apartment/Condominium	468	18.6
Terrace house/twin house	1030	41
Bungalow/Village house	1041	40.4
Does your house have a lot of plants or vegetation		
None	147	5.9
Low	911	36.3
Moderate	676	26.9
A lot	778	31
Your living area		
Urban	864	34.4
Suburban	801	31.9
Rural	847	33.7

### Dengue fever knowledge

**Fig. 2:**
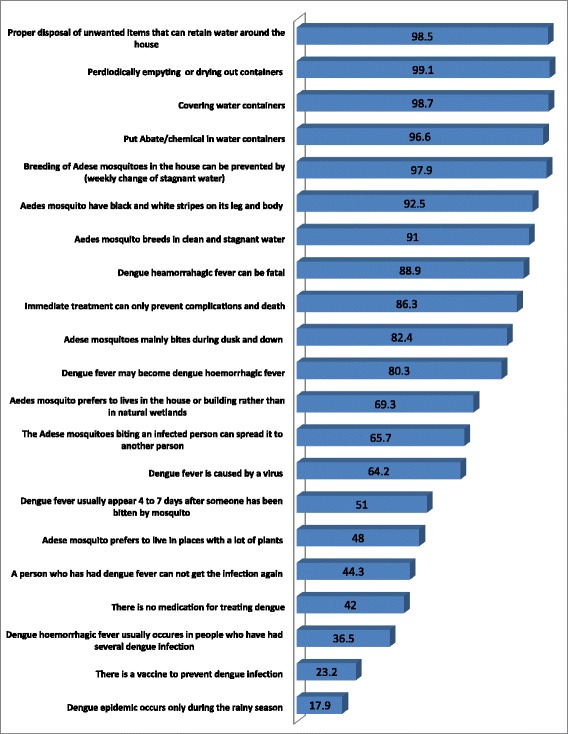
Percentage of correct responses to dengue knowledge items

More than half of participants (*n* =1612, 64.2 %) knew a virus causes that dengue fever. Many of the participants (*n* =2287, 91 %) reported that Aedes mosquito breeds in clean and stagnant water. Most of the participants (*n* = 2330; 92.8 %) were aware that the *Aedes aegypti* mosquito has black and white stripes on its legs and body. More than one-third of the participants (*n* = 917; 36.5 %) indicated that dengue haemorrhagic fever (DHF) usually occurs in people who had several dengue infections. Less than half of participants (*n* = 1205; 48.0 %) reported that the *Aedes spp.* mosquito could live in places with a lot of plants. About half of participants (*n* = 1282; 51.0 %) indicated that dengue usually appears four to seven days after a mosquito has bitten someone. Most participants (*n* = 2261; 90.0 %) were aware that fever is a symptom of dengue infections. However, only 39.2 % (*n* = 985) of participants knew that pain in the eyes is a symptom of dengue. Many of the participants (*n* = 1930; 76.8 %) were aware that there is no vaccine to prevent dengue infection (Fig. 2). The mean total dengue fever knowledge score for the overall sample was 27.49 (SD ± 8.34), out of a possible score of 42.

### Health beliefs

Socio-demographic characteristics were not significantly associated with the perceived severity of dengue scores. Many participants (71.4 %) had scores below 5 (out of possible 10 score) for perceived susceptibility of dengue fever. Marital status and gender were significantly associated to perception of susceptibility of dengue fever. A significantly higher proportion of married participants (30.3 %) had scores above 5 for perceived susceptibility of dengue fever compared to single participants (25.3 %), *χ*2 = 5.90, df 1, *p* < 0.05. A significantly higher proportion of male participants (31.9 %) had scores above 5 for perceived susceptibility of dengue fever compared to female participants (27.3 %), *χ*2 = 5.40, df 1, *p* < 0.05. More than two-third of participants (79.7 %) had scores above 5 for perceived barriers to prevent dengue fever. All socio-demographic characteristics were not significantly associated with the perceived barriers to prevent dengue fever scores. More than half (69.5 %) indicated they agreed or strongly agreed that they lacked the self-efficacy to prevent dengue. Significantly higher proportions of female participants (72.7 %) indicated that they agreed or strongly agreed to lacking self-efficacy in preventing dengue compared to male participants (61.8 %), *χ*2 = 5.40, df 1, *p* < 0.05.

### Factors associated with perceived acceptance of the dengue home test kit

Of the total contacted participants, only 152 (6.1 %) reported that they had heard or knew of the possible availability of the dengue home test kit. When asked if they would consider using a dengue home test kit if the kit became available, less than half of the all participants in this study (*n* = 1126, 44.8 %) answered “yes”. When all participants were asked if they would be able to pick their finger to do self-testing, only 28.4 % answered “yes”.

As shown in Table 2, those who were single and participants with a tertiary educational level were more likely to consider using a home self-test kit for dengue if the kit were available. Participants with higher mean total dengue fever knowledge scores (23–44) had significantly higher proportions of using the self-testing dengue kit for dengue if the kit were available (47.6 %, *n* = 816) compared to lower total dengue fever knowledge scores (range 0-22; 38.9 %, *n* = 310). Among those willing to use the dengue test kit, 45.5 % of them have the perceived severity of dengue fever score of 6–10 compared to only 38.7 % with score of 0–5. A total of 48.6 % of those willing to use dengue test kit have the perceived severity score of 6–10, compared to only 43.9 % with score of 0–5. In the multivariate logistic regressions analysis, when all the significant associations in the univariate analyses were entered into the model, the results showed that participants with Malay [odd ratio (OR) 0.55; 95 % confidence interval (CI) 0.33–0.93; *p* = 0.001], Chinese [OR: 0.23; 95 % CI: 0.13–0.41; *p* = 0.01] and Indian [OR:0.35; 95 % CI: 0.19–0.64; *p* = 0.02] ethnicity indicated that they were less likely, compared to other ethnicities, to use a home self-testing dengue kit for dengue if the kit were available. Participants with primary educational levels were less likely than participants with a tertiary educational level to use a home self-testing dengue kit for dengue if the kit were available [OR: 0.65; 95 % CI: 0.43–0.89; *p* = 0.02]. Participants with lower perceived barriers to dengue prevention (level of barriers 0–5) were less likely (OR: 0.67, 95 % CI, 0.53–0.85, *p* < 0.001) to use a home self-testing dengue kit for dengue if the kit were available compared to the reference level of barriers 6–10. Participants with lower total dengue fever knowledge scores (range 0–22) were less likely to use a home self-testing dengue kit for dengue if the kit were available [OR: 0.75; 95 % CI: 0.61–0.91; *p* = 0.001] compared to the reference group (participants with higher total dengue fever knowledge scores; range 23–44).

**Table 2:** Factors associated with willingness to use self-testing kit for dengue if the kit were available (*N* = 2512)

Socio-demographic variables	Willingness to use dengue home test kit				Logistic regression model (*N* = 2512) OR (95 % CI) for yes vs. no
	Frequency	Yes (1126)	No (1386)	*p*	
	N (%)	N (%)	N (%)		
Age (years)					
18–30	925(36.8)	458(49.5)	467(50.5)		1.32(0.91,1.92)
31–45	562(22.4)	238(42.3)	324(57.7)	0.011	1.03(0.80,1.33)
46 or above	1025(40.8)	430(42.0)	595(58.0)		1
Gender					
Male	717(28.5)	332(40.3)	385(53.7)	0.071	-
Female	1795(64.3)	794(44.2)	1001(55.8)		
Marital status					
Single	766(35.7)	392(51.2)	374(48.8)	0.021	1.09(0.70,1.43)
Ever married	1381(64.3)	610(44.2)	771(55.8)		1
Ethnicity					
Malay	1394(55.5)	713(51.1)	681(48.9)	0.001	0.55(0.33,0.93)*
Chinese	771(30.7)	247(32.0)	524(68.0)		0.23(0.13,0.41)
Indian	249(9.9)	101(40.6)	148(59.4)		0.35(0.19,0.64)
Others	98(3.9)	65(66.3)	33(33.7)		1
Religion					
Muslim	1477(58.8)	757(51.30)	720(48.7)	0.066	
Christian	152(6.1)	55(36.2)	97(63.8)		
Buddhist	515(20.5)	160(32.2)	349(67.8)		
Others	368(14.6)	148(40.2)	220(59.8)		
Highest educational level					
Primary school	396(15.8)	155(39.1)	241(60.9)	0.001	0.65(0.43,0.89)*
Secondary school	1292(51.4)	570(44.1)	722(55.9)		0.73(0.57,0.90)
Tertiary	824(32.8)	401(48.7)	423(51.3)		1
Occupation					
Skilled worker	667(26.6)	299(44.8)	368(55.2)	0.051	-
Non-skilled worker	396(15.8)	169(42.7)	227(57.3)		-
Unemployed	1449(57.7)	658(45.4)	791(54.6)		-
Monthly average household income					
≤ RM2000	790(31.4)	380(48.1)	410(51.9)	0.071	-
> RM2000	1722(68.6)	746(43.3)	976(56.7)		-
Dengue experience					
Yes (once)	148(5.9)	77(52)	71(48.0)	0.064	-
No	2364(94.1)	1049(44.4)	1315(55.6)	-	
Health beliefs					
Perceived severity of dengue fever					
0–5	201(8.0)	76(37.8)	125(62.2)	0.002	0.90(0.64,1.27)
6–10	2311(92.0)	1050(45.4)	1261(54.6)		1
Perceived susceptibility to dengue fever					
0–5	1793(71.4)	805(44.9)	988(55.1)	0.052	-
6–10	719(28.6)	321(44.6)	398(55.4)		-
Perceived barriers to dengue prevention					
0–5	2002(79.7)	878(43.9)	1124(56.1)	0.031	0.67(0.53,0.85)*
6–10	510(20.3)	248(48.6)	262(51.4)		1
Self-Efficiency					
Agree	1747(69.5)	776(44.4)	971(55.6)	0.061	-
Disagree	765(31.5)	350(45.8)	415(54.2)		
Dengue fever knowledge score					
0–22	797(31.7)	310(38.9)	487(61.6)	0.001	0.75(0.61,0.91)*
23–44	1715(68.3)	816(47.6)	899(52.4)		1

Note: Not all subtotals add up to the total of 2,512 owing to missing values

Hosmer and Lemeshow test, *χ*2 (8) = 10.48, *p* = 0.67; Cox & Snell R2 = 0.511; Nagelkerke R2 = 0.685

*p < 0.001

### Characteristics of the test kits

As shown in Table 3, the following 5 characteristics influenced participants’ overall acceptance of the dengue home test kit. The majority (*n* = 114, 63.7) mentioned ease of use and easy to understand instructions, followed by the price of the test kit, the accuracy of the test, recommendation by healthcare providers and ease of accessibility. Participants that noted ease of use and easy to understand instructions as important to the use of the dengue home test kit [OR: 2.42; 95 % CI: 1.76–3.32; *p* = 0.001] were more likely to use the home test kit if the kit were available (Table 3).

**Table 3:** Characteristics of the test kits that influence the willingness to use dengue home test kit *N* = 2512)

	Total	Use dengue home test kit (*N* = 1126)	Do not use dengue home test kit (*N* = 1386)	*P*	Logistic regression model (*N* = 2512) OR (95 % CI) for use *vs.* do not use
	N	N (%)	N (%)		
Easy to obtain					
Yes	206	88(42.7)	118(57.3)	0.06	-
No	2306	1038(45)	1268(55)		-
Easy to use and easy to understand instructions					
Yes	179	114(63.7)	65(36.3)	0.02	2.42(1.76,3.32)*
No	2333	1012(43.4)	1321(56.6)		1
Accuracy of test kit					
Yes	229	105(45.9)	124(54.1)	0.51	-
No	2283	1021(44.7)	1262(55.3)		-
Price					
Yes	277	149(53.8)	128(46.2)	0.01	1.60(1.25,2.07)*
No	2235	977(43.7)	1258(56.3)		1
Receiving health care provider recommendations					
Yes	2035	914(44.9)	1121(55.1) 0.07		-
No	477	212(44.4)	265(55.6)		-

Logistic regression model of predictors of Use vs. Do not use; Hosmer and Lemeshow test, *χ* ^2^ (2) = 41.76, *p* = 0.72; Cox & Snell R^2^ = 0.016; Nagelkerke R^2^ = 0.022

*p < 0.001

## Discussion

Perceived acceptance of self-testing for dengue if it becomes available is moderate, with less than half of the participants expressing their intention to use the kit. With the expectation that the dengue home test kit perhaps one day soon will be made available, efforts to raise public acceptance of the test kit is essential. In the current study, participants 16 to 30 years old had a higher proportion of perceived acceptance of the dengue home test kit compared to those participants who were more than 30 years old. Our findings suggest the attitude of participants on the perceived acceptance toward use of the dengue test kit among participants more than 30 years old could reshape our approach on message delivery to this vulnerable target population. Single participants had a higher proportion of perceived acceptance of the dengue home test kit compared to married participants. This may be due to the fact that single participants had a better understanding of information provided about dengue fever than did married participants.

Highly educated participants tended to have a higher level of acceptance of test kits; this suggests that perceived acceptance of the dengue home test kit is relatively low among people with lower education, thus more effective education programs related to population awareness of the dengue home test kit needs to be implemented among people with low education levels especially in large cities with high population density where dengue dominates. Participants with a Malay ethnicity had a higher proportion of perceived acceptance of the dengue home test kit if the kit became available compared to those Chinese and Indian participants. Disparities across the three main ethnic groups (Malay, Chinese, and Indian) in use of dengue home test kit if available are not known and this warrants qualitative study for further investigation.

In the current study, participants with higher knowledge of dengue fever had a significantly higher proportion on the perceived acceptance of dengue home test kit if available. An explanation for the positive association could be that people with more knowledge about dengue fever are more aware of benefits and advantages of acceptance of dengue home test kit. Our study suggests that knowledge about dengue fever is an important factor in decisions to order and use the dengue home test kit; it might, therefore, be worthwhile trialing targeted educational interventions to improve knowledge about dengue fever. It is recommended that dengue educational programs add a focus on increasing knowledge of the importance of early diagnosis of dengue and benefit from the acceptance of a dengue home test kit if one should become available [16, 17].

In addition, answers to the research question on which factors influence the decision to use the home test kit indicated that ease of usability and easy to understand instructions, cost and accuracy of the test kit appeared to be the most important determining factors. Therefore, it is essential for providers to determine reasonable pricing of the test kit. Participants also suggest that they want to be assured of the accuracy of the dengue home test kit. It is also important that the dengue home test kit is easy to use, with easy to understand instructions [18].

Finally, the multivariate analysis findings from our study provide a base for creating educational and health messages for health behavioral change interventions based on the HBM constructs Our study revealed that higher perceived barriers to dengue prevention was associated with a higher proportion of perceived acceptance of the dengue home test kit. The reason why participants with higher perceived barriers of dengue prevention had a higher proportion of perceived acceptance of the dengue home test kit compared to participants’ with lower perceived barriers is not known and therefore future investigation is essential. While multivariate analysis was unable to determine the association between perceived severity of dengue fever and perceived acceptance of the dengue home test kit, univariate analyses showed that, the higher perceived severity of dengue fever, the higher perceived acceptance of the dengue home test kit.

Among the primary limitation of the study is that the computer-assisted telephone survey only included households with fixed line telephones; therefore, households without a telephone line (which are more likely to be from socio-economically disadvantaged groups) were under-represented. A second limitation concerns the fact that we only used a self-report, telephone interview method, which may contribute to information bias due to self-reporting, and may have influenced some of the findings.

## Conclusion

In conclusion, perceived acceptance of self-testing for dengue if it is available is poor among the community, with less than half of participants expressing their intention to use the kit. The significant factors that correlate to use of the dengue home test kit were perceived barriers, knowledge and education level. This suggests the need to enhance the acceptance of the dengue home test kit among participants with a lower educational level and those with poor knowledge about dengue prevention. Findings revealed a number of important issues which need to be addressed before such tests are introduced, including the following: provide dengue home test kits with reasonable prices, ensure ease of use and providing information about the accuracy of the test kit.

## Additional files

Additional file 1: Questionnaire. (DOC 405 kb) Additional file 2: Dengue Public Nationwide. (SAV 1.07 mb)
