# Supplementary material for: Nationwide study of factors associated with public’s willingness to use home self-test kit for dengue fever in Malaysia
Source: BMC Public Health. 2016 Aug 12;16:780. doi: 10.1186/s12889-016-3409-y (PMC4982416; doi:10.1186/s12889-016-3409-y)
Supplement: Additional file 1: — Questionnaire. (DOC 405 kb) [file 12889_2016_3409_MOESM1_ESM.doc]

*CONFIDENTIAL*

ID

**KNOWLEDGE, ATTITUDE AND PRACTICES REGARDING DENGUE FEVER AMONG MALAYSIAN POPULATION**

# **Section A**

# DENGUE EXPERIENCE

A. Following are statements regarding dengue experiences.

| A.1 | Have you ever had dengue fever? | | | | | [ 1 ] Ya/Yes [ 2 ] Tidak/No | | | | |
| --- | --- | --- | --- | --- | --- | --- | --- | --- | --- | --- |
| A.2 | How many times have you had dengue? | | | | | ____________ kali/times | | | | |
| A.3 | Have you been hospitalized because of dengue? | | | | | [ 1 ] Ya/Yes [ 2 ] No  Go to A.5 | | | | |
| A.4 | If you have, how many days hospitalized? | | | | | ____________ days | | | | |
| A.5 | Has anyone living in your household experienced dengue fever? | | | | | [ 1 ] Ya/Yes [ 2 ] Tidak/No  Terus ke A.8/Go to A.8 | | | | |
| A.6 | If Yes, how many people in your house has had dengue? | | | | | [ 1 ] Children ______ person  [ 2 ] Dewasa/Adult ______ person  [ 3 ] Old people (above 60 year) ______ person  [ 4 ] Nobody in the house has dengue | | | | |
| A.7 | How many days hospitalized? | | | | | | | | | |
|  | **CHILDREN** | | | **ADULT** | | | | **OLD PEOPLE** | | |
|  | 1^st^  ___days | 2^nd^  ___days | >2 person  _______ | 1^st^  ___days | 2^nd^  ___days | | >2 person  _______ | 1^st^  ___days | 2^nd^  ___days | >2 person  _______ |
| A.8 | Has anyone living in your neighborhood experienced dengue fever? | | | | | [ 1 ] Yes [ 2 ] No | | | | |
| A.9 | How would you rate the mosquito problem in your neighborhood? | | | | | [ 1 ] None  [ 2 ] Low  [ 3 ] Moderate  [ 4 ] Severe | | | | |
| A.10 | Frequency of mosquito fogging in your neighborhood | | | | | [ 1 ] None  [ 2 ] Rarely  [ 3 ] Occasionally  [ 4 ] Often | | | | |
| A.11 | Do you often travel or been in an area where dengue has been reported? | | | | | [ 1 ] Yes [ 2 ] No  [ 3 ] Not sure | | | | |

# **Section B**

# SEVERITY PERCEPTION

B. Following are statements regarding your perceived severity (belief of how serious a condition and its consequences are ) regarding dengue infection.

|  | Statement | Rank | | | | | | | | | | | | | |
| --- | --- | --- | --- | --- | --- | --- | --- | --- | --- | --- | --- | --- | --- | --- | --- |
| B.1 | In your opinion, do you think dengue is dangerous?Please state from scale 1 (not dangerous) to 10 (very dangerous) | 1  Not dangerous | 2 | 3 | | 4 | 5 | | 6 | 7 | | 8 | 9 | | 10  Very dangerous |
|  | Statement | | | | Strongly agree | | | Agree | | | Disagree | | | Strongly disagree | |
| B.1.1 | Dengue does not always lead to death | | | | [ 1 ] | | | [ 2 ] | | | [ 3 ] | | | [ 4 ] | |
| B.1.2 | Dengue infection is only dangerous to children | | | | [ 1 ] | | | [ 2 ] | | | [ 3 ] | | | [ 4 ] | |
| B.1.3 | Dengue infection is only dangerous to old people | | | | [ 1 ] | | | [ 2 ] | | | [ 3 ] | | | [ 4 ] | |

Following are statements regarding your perceived susceptibility of dengue infection.

| B.2 | Untuk orang yang pernah kena denggi, apakah kemungkinan/risiko anda dijangkiti lagi?/For those who have had dengue, how likely are you to get it again (risk of getting dengue)? |  | 1 | 2 | 3 | | 4 | 5 | | 6 | 7 | | 8 | 9 | | 10 |
| --- | --- | --- | --- | --- | --- | --- | --- | --- | --- | --- | --- | --- | --- | --- | --- | --- |
|  |  | Very unlikely | |  | | | | | | | | | | Highly likely | | |
|  | Statement | | | | | Strongly agree | | | Agree | | | Disagree | | | Strongly disagree | |
| B.2.1 | I am unlikely to get dengue because there are no dengue fever cases in my neighborhood | | | | | [ 1 ] | | | [ 2 ] | | | [ 3 ] | | | [ 4 ] | |
| B.2.2 | I am unlikely to get dengue because I carry out proper mosquito prevention practices | | | | | [ 1 ] | | | [ 2 ] | | | [ 3 ] | | | [ 4 ] | |
| B.2.3 | I am unlikely to get dengue because my body is strong | | | | | [ 1 ] | | | [ 2 ] | | | [ 3 ] | | | [ 4 ] | |
| B.2.4 | I am unlikely to get dengue infection because I already had it before | | | | | [ 1 ] | | | [ 2 ] | | | [ 3 ] | | | [ 4 ] | |
| B.2.5 | I am unlikely to get dengue because dengue is likely to strike rural folk living near forests or swamps | | | | | [ 1 ] | | | [ 2 ] | | | [ 3 ] | | | [ 4 ] | |
| B.2.6 | I am unlikely to get denggi because my house surrounding is clean | | | | | [ 1 ] | | | [ 2 ] | | | [ 3 ] | | | [ 4 ] | |

Following are statements regarding perceived barriers to dengue prevention.

| B. 3 | From scale 1 (no barriers) to 10 (high barrier) how do you rate the barriers you face to prevent dengue: |  | 1 | 2 | 3 | | 4 | 5 | | 6 | 7 | | 8 | 9 | | 10 |
| --- | --- | --- | --- | --- | --- | --- | --- | --- | --- | --- | --- | --- | --- | --- | --- | --- |
|  |  | No barriers at all | |  | | | | | | | | | | High barrier level | | |
|  | Statement | | | | | Strongly agree | | | Agree | | | Disagree | | | Strongly disagree | |
| B.3.1 | Lack of community participation in taking preventive measures against dengue | | | | | [ 1 ] | | | [ 2 ] | | | [ 3 ] | | | [ 4 ] | |
| B.3.2 | Lack of self-efficacy in taking preventive measures against dengue  Contoh: | | | | | [ 1 ] | | | [ 2 ] | | | [ 3 ] | | | [ 4 ] | |
| B.3.3 | Lack of preventive measures from authorities | | | | | [ 1 ] | | | [ 2 ] | | | [ 3 ] | | | [ 4 ] | |

# **Section C**

# KNOWLEDGE RELATED TO DENGUE

C. The following are dengue knowledge test questions, please answer “True”, “False” or “Don’t know”.

| Statements | | True | | False | | Don’t know |
| --- | --- | --- | --- | --- | --- | --- |
| Knowledge about dengue and Aedes mosquito | | | | | | |
| C.1 | Dengue is transmitted by mosquito | [ 1 ] | | [ 2 ] | | [ 3 ] |
| C.2 | The dengue virus is transmitted by Aedes mosquito | [ 1 ] | | [ 2 ] | | [ 3 ] |
| C.3 | Dengue fever is caused by a **virus** | [ 1 ] | | [ 2 ] | | [ 3 ] |
| C.4 | Dengue fever may become **Dengue Haemorrhagic Fever** | [ 1 ] | | [ 2 ] | | [ 3 ] |
| C.5 | **Dengue haemorrhagic fever** can be fatal | [ 1 ] | | [ 2 ] | | [ 3 ] |
| C.6 | Dengue Haemorrhagic Fever usually occurs in people who have had several dengue infection | [ 1 ] | | [ 2 ] | | [ 3 ] |
| C.7 | Aedes mosquitos have **black and white stripes on its leg and body** | [ 1 ] | | [ 2 ] | | [ 3 ] |
| C.8 | Aedes mosquito breeds in clean and stagnant water | [ 1 ] | | [ 2 ] | | [ 3 ] |
| C.9 | Aedes mosquito prefers to live in the house or building rather than in natural wetlands | [ 1 ] | | [ 2 ] | | [ 3 ] |
| C.10 | Aedes mosquito prefers to live in places with a lot of plants | [ 1 ] | | [ 2 ] | | [ 3 ] |
| C.11 | Aedes mosquitoes mainly **bite during dusk and dawn** | [ 1 ] | | [ 2 ] | | [ 3 ] |
| Knowledge about the transmission of dengue | | | | | | |
| C.12 | The Aedes mosquitoes biting an infected person can spread it to another person | [ 1 ] | | [ 2 ] | | [ 3 ] |
| C.13 | Dengue fever usually appear **4 to 7** **days** after someone has been bitten by mosquito | [ 1 ] | | [ 2 ] | | [ 3 ] |
| C.14 | Dengue disease can be transmitted from an infected person by:   1. Touching 2. Air 3. Body fluid (saliva,sweat, semen) 4. Blood | [ 1 ]  [ 1 ]  [ 1 ]  [ 1 ] | | [ 2 ]  [ 2 ]  [ 2 ]  [ 2 ] | | [ 3 ]  [ 3 ]  [ 3 ]  [ 3 ] |
| C.15 | Aedes mosquitoes’ eggs can contain the dengue virus | [ 1 ] | | [ 2 ] | | [ 3 ] |
| C.16 | A person who has had dengue fever cannot get the infection again | [ 1 ] | | [ 2 ] | | [ 3 ] |
| C.17 | Dengue epidemic occurs only during the rainy season | [ 1 ] | | [ 2 ] | | [ 3 ] |
| Knowledge about prevention | | | True | | False | Don’t know |
| C.18 | Breeding of Aedes mosquitoes in the house can be prevented by:   1. **weekly change of stagnant** **water** (pet bowls, flower pots or vases, etc) in and around the house 2. **Put Abate/chemical** in water containers 3. **covering** water containers 4. periodically **emptying or drying out containers** (old tires, trash cans, coconut shell etc) that retain water in and around the house 5. **proper disposal** of items that can retain water (old tires, trash cans, coconut shell etc) around the house | | [ 1 ]  [ 1 ]  [ 1 ]  [ 1 ]  [ 1 ] | | [ 2 ]  [ 2 ]  [ 2 ]  [ 2 ]  [ 2 ] | [ 3 ]  [ 3 ]  [ 3 ]  [ 3 ]  [ 3 ] |
| Knowledge on signs and symptoms | | | | | | |
| **Signs and symptoms of dengue fever** | | True | | False | | Don’t know |
| C.19 | The high fever for 5 to 6 days | [ 1 ] | | [ 2 ] | | [ 3 ] |
| C.20 | Chills | [ 1 ] | | [ 2 ] | | [ 3 ] |
| C.21 | Rash | [ 1 ] | | [ 2 ] | | [ 3 ] |
| C.22 | Pain in the eyes | [ 1 ] | | [ 2 ] | | [ 3 ] |
| C.23 | Joint pain | [ 1 ] | | [ 2 ] | | [ 3 ] |
| C.24 | Headache | [ 1 ] | | [ 2 ] | | [ 3 ] |
| C.25 | Stomach ache | [ 1 ] | | [ 2 ] | | [ 3 ] |
| C.26 | Nausea and vomiting | [ 1 ] | | [ 2 ] | | [ 3 ] |
| **Signs and symptoms of Dengue Haemorrhagic Fever (DHF)** | | | | | | |
| C.27 | red or purple spots under the skin | [ 1 ] | | [ 2 ] | | [ 3 ] |
| C.28 | Bleeding in the nose | [ 1 ] | | [ 2 ] | | [ 3 ] |
| C.29 | Bleeding in gums | [ 1 ] | | [ 2 ] | | [ 3 ] |
| C.30 | Blood in stool | [ 1 ] | | [ 2 ] | | [ 3 ] |
| C.31 | Blood in urine | [ 1 ] | | [ 2 ] | | [ 3 ] |
| C.32 | Shortness of breath | [ 1 ] | | [ 2 ] | | [ 3 ] |
| C.33 | Diziness or fainting | [ 1 ] | | [ 2 ] | | [ 3 ] |
| Knowledge about treatment, curability and precaution measures for people infected with dengue | | | | | | |
| Statements | | True | | False | | Don’t know |
| C.34 | There is no medication for treating dengue | [ 1 ] | | [ 2 ] | | [ 3 ] |
| C.35 | Immediate treatment can only prevent complications and death | [ 1 ] | | [ 2 ] | | [ 3 ] |
| C.36 | There is a vaccine to prevent dengue infection | [ 1 ] | | [ 2 ] | | [ 3 ] |

# **Section D**

# PRACTICES REGARDING DENGUE PREVENTION

D. Following are statements regarding your dengue prevention practices. Please answer “Not at all”, “Rarely”, “Sometimes”, “Often” or “Not applicable”.

| Statements | | | Not at all | Rarely | Sometimes | Often | Not applicable | |
| --- | --- | --- | --- | --- | --- | --- | --- | --- |
| Prevention of mosquito breeding | | |
| D.1 | **Cover** all water containers used for storing water in or outside the house | | [ 1 ] | [ 2 ] | [ 3 ] | [ 4 ] | [ 5 ] | |
| D.2 | Change **stored water** i.e water in water tank, pails, bath tub, flower vases, money plant, **plates that support plant pots, refrigerator drip tray, plastic container on the bottom of kitchen cabinet/table legs (for controlling ants), etc.** | | [ 1 ] | [ 2 ] | [ 3 ] | [ 4 ] | [ 5 ] | |
| D.3 | **Put Abate or chemical** in water storage containers | | [ 1 ] | [ 2 ] | [ 3 ] | [ 4 ] | [ 5 ] | |
| D.4 | **Examine for mosquito larvae** in containers for storing water, eg. pails, flower vases, water in money plant, plates that support plant pots, refrigerator drip tray, plastic container on the bottom of kitchen cabinet/table legs, etc. | | [ 1 ] | [ 2 ] | [ 3 ] | [ 4 ] | [ 5 ] | |
| D.5 | **Clear out debris** that may **block water flow** in drain or **roof** gutters | | [ 1 ] | [ 2 ] | [ 3 ] | [ 4 ] | [ 5 ] | |
| D.6 | **Proper disposal of items** **that can collect rain water** (i.e. bucket, discarded item such as cans, cups,bottles, food container, linolium, old tyres etc.) | | [ 1 ] | [ 2 ] | [ 3 ] | [ 4 ] | [ 5 ] | |
|  | Statements | | Not at all | Rarely | Sometimes | Often | Not applicable | |
| D.7 | Proper **disposal of household garbage** | | [ 1 ] | [ 2 ] | [ 3 ] | [ 4 ] | [ 5 ] | |
| D.8 | **Clean up surrounding house area** | | [ 1 ] | [ 2 ] | [ 3 ] | [ 4 ] | [ 5 ] | |
| D.9 | Take mosquito preventive measures before going on **long holidays** such as covering all water containers, emptying water containers etc. | | [ 1 ] | [ 2 ] | [ 3 ] | [ 4 ] | [ 5 ] | |
| Prevention of mosquito bites | | | | | | | | |
| D.10 | | Sleep in mosquito net or have mosquito screens on windows | [ 1 ] | [ 2 ] | [ 3 ] | [ 4 ] | | [ 5 ] |
| D.11 | | Use mosquito coil, electric mosquito mat,liquid vaporizer, mosquito bulb, or mosquito trap | [ 1 ] | [ 2 ] | [ 3 ] | [ 4 ] | | [ 5 ] |
| D.12 | | dark places with an insecticidal spray | [ 1 ] | [ 2 ] | [ 3 ] | [ 4 ] | | [ 5 ] |
| D.13 | | Use mosquito repellent on body | [ 1 ] | [ 2 ] | [ 3 ] | [ 4 ] | | [ 5 ] |
| D.14 | | Avoid dark areas in the home where there is no light and no wind | [ 1 ] | [ 2 ] | [ 3 ] | [ 4 ] | | [ 5 ] |
| D.15 | | Wear long-sleeved shirts and pants to avoid mosquito bites | [ 1 ] | [ 2 ] | [ 3 ] | [ 4 ] | | [ 5 ] |
| D.16 | | Wear bright color clothes to avoid mosquito bites | [ 1 ] | [ 2 ] | [ 3 ] | [ 4 ] | | [ 5 ] |
| Prevention of dengue transmission | | | | | | | | |
| D.17 | | Take measures to prevent mosquitoes from biting a dengue patient | [ 1 ] | [ 2 ] | [ 3 ] | [ 4 ] | | [ 5 ] |

# **Section E**

# GENERAL PRACTICES REGARDING DENGUE TREATMENT

The following are the **treatment/treatment source for children (under 12 years old),adults and old aged people in your household suspected or diagnosed with dengue fever.**

| Statements | | Your children | | | | Any adults in the household | | | | Old aged people | | | |
| --- | --- | --- | --- | --- | --- | --- | --- | --- | --- | --- | --- | --- | --- |
| Treatment seeking for children, adults and old aged people in your household when they experience dengue like symptoms | | Yes | No | Not applicable | | Yes | No | Not applicable | | Yes | No | Not applicable | |
|  |  |  |  | Never suspected or diagnosed | No children |  |  | Never suspected or diagnosed | No adult |  |  | Never suspected or diagnosed | No old people |
| E.1 | See a doctor | [1] | [2] | [3] | [4] | [5] | [6] | [ 7 ] | [ 8 ] | [ 9 ] | [ 10] | [ 11 ] | [ 12 ] |
| E.2 | Get medicine from pharmacy | [1] | [2] | [3] | [4] | [5] | [6] | [ 7 ] | [ 8 ] | [ 9 ] | [ 10] | [ 11 ] | [ 12 ] |
| E.3 | Use traditional remedies (i.e papaya leaf juice, tumeric) | [1] | [2] | [3] | [4] | [5] | [6] | [ 7 ] | [ 8 ] | [ 9 ] | [ 10] | [ 11 ] | [ 12 ] |
| E.4 | Traditional healers (i.e. sinseh, bomoh, pawang, dukun, alternative practitioner or road side medicine man) | [1] | [2] | [3] | [4] | [5] | [6] | [ 7 ] | [ 8 ] | [ 9 ] | [ 10] | [ 11 ] | [ 12 ] |
| E.5 | Self-treatment (i.e. sponge bathing to reduce fever) | [1] | [2] | [3] | [4] | [5] | [6] | [ 7 ] | [ 8 ] | [ 9 ] | [ 10] | [ 11 ] | [ 12 ] |
| E.6 | No treatment (do nothing) | [1] | [2] | [3] | [4] | [5] | [6] | [ 7 ] | [ 8 ] | [ 9 ] | [ 10] | [ 11 ] | [ 12 ] |

Note: Not applicable = no experience of dengue symptom or no children/adults/old aged people in household

# **Section F**

# DENGUE TEST KIT

F.The following are statements regarding attitudes towards self testing for dengue.

| F.1 | Have you heard of home self testing kit for dengue infection? | [ 1 ] Yes  [ 2 ] No |
| --- | --- | --- |
| F.2 | Would you consider using a home self test kit ? | [ 1 ] Yes  [ 2 ] No  [ 0 ] Not sure |
| F.3 | Would you be able to prick your finger to do self testing? | [ 1 ] Yes, I would prick myself  [ 2 ] No, I would let someone else prick my finger  [ 2.1 ] Who? Please state: _________________________________________  [ 3 ] Tidak pasti/ Not sure |
| F.4 | Among the following 5 characteristics, which one is the most important for you? | Please answer only one:  [ 1 ] Easy to obtain  [ 2 ] Easy to use and instruction easy to understand  [ 3 ] Accuracy of test kit  [ 4 ] Price  [ 5 ] Recommended by health care providers (eg. doctors, nurses)  [ 6 ] Others:_____________________________ |
| F.5 | Reasonable price per kit is RM__________ | |

# **Section G**

# GENERAL INFORMATION

| G.1 | Age | ____________ Years old |
| --- | --- | --- |
| G.2 | Gender | [ 1 ] Male  [ 2 ] Female |
| G.3 | Ethnicity | [ 1 ] Malay  [ 2 ] Chinese  [ 3 ] Indian  [ 4 ] Bumiputera Sabah/Sarawak  [ 6 ] Aborigine :_________________________________  [ 5 ] Others, please specify : ___________________________________________________ |
| G.4 | Religion | [ 1 ] Muslim  [ 2 ] Buddhist  [ 3 ] Taoist  [ 4 ] Hindu  [ 5 ] Christian  [ 6 ] Others, please specify :  _________________________________________ |
| G.5 | Highest educational level | [ 1 ] No formal education  [ 2 ] Primary school  [ 3 ] Secondary school  [ 4 ] Tertiary |
| G.6 | Occupation | Please specify: _______________________________________________  Category:  [ 1 ] Professional and managerial  [ 2 ] Skilled worker  [ 3 ] Non-skilled worker  [ 4 ] Student  [ 5 ] Housewife  [ 6 ] Retired  [ 7 ] Others: ____________________________ |
| G.7 | How many people living in the house | [ 1 ] Adult________________________________________  [ 2 ] Children__________________________________  [ 3 ] Old people (60 years above)_________________ |
| G.8 | Monthly average household income | RM_____________ month  [ 1 ] Below RM1000  [ 2 ] RM1001 - RM2000  [ 3 ] RM2001 - RM3000  [ 4 ] RM3001 - RM4000  [ 5 ] RM4001 - RM5000  [ 6 ] Above RM5000 |
| G.9 | Type of house | [ 1 ] Flat/ Apartment/ Condominium (High rise houses)  [ 2 ] Terrace house/twin house  [ 3 ] Bungalow/ Village house |
| G.10 | Does your house have a lot of plants or vegetation? | [ 1 ] None  [ 2 ] Low  [ 3 ] Moderate  [ 4 ] A lot |
| G.11 | Your living area | [ 1 ] Urban  [ 2 ] Suburban  [ 3 ] Rural |
| G.12 | State | [ 1 ] WP Kuala Lumpur  [ 2 ] WP Putrajaya  [ 3 ] Selangor  [ 4 ] Johor  [ 5 ] Negeri Sembilan  [ 6 ] Melaka  [ 7 ] Pahang  [ 8 ] Perak  [ 9 ] Kedah  [ 10 ] Perlis  [ 11 ] Terengganu  [ 12 ] Kelantan  [ 13 ] Sabah /WP Labuan  [ 14 ] Sarawak |
